# Amyloid beta in nasal secretions may be a potential biomarker of Alzheimer’s disease

**DOI:** 10.1038/s41598-019-41429-1

**Published:** 2019-03-21

**Authors:** Young Hyo Kim, Sang-Myung Lee, Sungbo Cho, Ju-Hee Kang, Yang-Ki Minn, Hyelim Park, Seong Hye Choi

**Affiliations:** 1 Departments of Otorhinolaryngology-Head and Neck Surgery, Inha University School of Medicine, Incheon, 22332 South Korea; 2 Department of Chemical Engineering, Kangwon National University, Chuncheon, 24341 South Korea; 3 Department of Electronic Engineering, Gachon University, Seongnam, 13120 South Korea; 4 Department of Pharmacology, Inha University School of Medicine, Incheon, 22212 South Korea; 5 Department of Neurology, Hallym University Kangnam Sacred Heart Hospital, Hallym University College of Medicine, Seoul, 07441 South Korea; 6 Department of Neurology, Inha University School of Medicine, Incheon, 22332 South Korea

## Abstract

We investigated the level of amyloid beta (Aβ) in nasal secretions of patients with Alzheimer’s disease dementia (ADD) using interdigitated microelectrode (IME) biosensors and determined the predictive value of Aβ in nasal secretions for ADD diagnosis. Nasal secretions were obtained from 35 patients with ADD, 18 with cognitive decline associated with other neurological disorders (OND), and 26 cognitively unimpaired (CU) participants. Capacitance changes in IMEs were measured by capturing total Aβ (ΔC_tAβ_). After 4-(2-hydroxyethyl)-1-piperazinepropanesulfonic acid (EPPS) was injected, additional capacitance changes due to the smaller molecular weight Aβ oligomers disassembled from the higher molecular weight oligomeric Aβ were determined (ΔC_oAβ_). By dividing two values, the capacitance ratio (ΔC_oAβ_/ΔC_tAβ_) was determined and then normalized to the capacitance change index (CCI). The CCI was higher in the ADD group than in the OND (p = 0.040) and CU groups (p = 0.007). The accuracy of the CCI was fair in separating into the ADD and CU groups (area under the receiver operating characteristic curve = 0.718, 95% confidence interval = 0.591–0.845). These results demonstrate that the level of Aβ in nasal secretions increases in ADD and the detection of Aβ in nasal secretions using IME biosensors may be possible in predicting ADD.

## Introduction

Alzheimer’s disease (AD) is the most common cause of dementia, as it accounts for 60 to 70% of the overall prevalence of dementia. AD is a neurodegenerative disorder characterized by an insidious onset and a progressive deterioration in cognition, functional ability, and behaviour^1^. Current therapies such as acetylcholinesterase inhibitors and the N-methyl-D-aspartate receptor antagonist memantine may provide relief with respect to symptoms, but they do not treat the underlying process of AD. Neuropathological hallmarks of AD include amyloid β (Aβ)-containing plaques and tau-containing neurofibrillary tangles, which are found throughout the brain^2^. These hallmarks have become the main target of current drug development. However, several drug candidates that target Aβ have not been successful in large, multicentre clinical trials^3–5^. It is now generally acknowledged that this is partly because a large proportion of clinically diagnosed patients show no evidence of amyloid pathology on positron emission tomography (PET) scans^5^. Recent advances in AD research also show that amyloid pathology begins 20–30 years before the clinical onset of AD^6^. These findings indicate the need for validated biomarkers in drug development and clinical practice.

In the last decade, remarkable progress has been achieved in the study of AD biomarkers. Biomarkers of Aβ plaques include cortical amyloid PET ligands^7–9^ and low cerebrospinal fluid (CSF) Aβ_42_^10,11^. Biomarkers of fibrillar tau include elevated CSF phosphorylated tau (p-tau) and cortical tau PET ligands^12,13^. Biomarkers of neurodegeneration or neuronal injury are CSF total tau (t-tau)^13^, ^18^fluorodeoxyglucose PET hypometabolism, and atrophy on magnetic resonance imaging (MRI)^14,15^. However, PET and MRI are expensive, and CSF biomarker analysis requires a lumbar puncture, which is an invasive procedure with potential adverse side effects such as headache and back pain. Interlaboratory variability of CSF biomarker analysis is also high. Recently, the results of studies of blood-based biomarkers of AD pathology such as plasma Aβ and tau have been reported^16–19^. Simple and inexpensive tests for blood-based biomarkers would be desirable due to their safety and minimal invasiveness. However, given the lack of consistency of most blood-based biomarkers across studies, validation in another cohort is needed, and other cost-effective and feasible methods for the early diagnosis of AD are needed.

In a recent study, older adults with olfactory dysfunction were more than twice as likely to develop dementia 5 years later, and more odour identification errors were associated with a greater probability of an interval dementia diagnosis^20^. It has been reported that in AβPP/PS1 transgenic mice, the deposition of Aβ began in the olfactory system and then spread to the hippocampus and cortex^21^. In experiments in rats, ventricle-injected isotope-labelled Aβ peptide (^125^I-Aβ40) was shown to be transported to the nasal cavity through a non-haematogenous pathway^22^. In another recent study, median levels of p-tau/t-tau ratios in the middle nasal meatus and in the olfactory cleft were significantly higher in AD cases than in controls, but the levels of Aβ_42_ and Aβ_40_ were near zero and were not different between AD cases and controls^23^. However, the methods used in the study may not have detected a very low level of Aβ in nasal secretions.

In this study, we investigated the level of Aβ in nasal secretions of patients with AD dementia (ADD) using interdigitated microelectrode (IME) methods and examined the predictive value of Aβ in nasal secretions for the diagnosis of ADD.

## Results

Nasal secretions were obtained from 35 patients with ADD, 18 patients with cognitive decline associated with other neurological disorders (OND), and 26 cognitively unimpaired (CU) participants. Table 1 presents the baseline demographic and clinical characteristics of the participants according to the groups. The participants in the CU group were younger than those in the ADD and OND groups, respectively. There was no significant difference in gender and education among the groups. The prevalence of Apolipoprotein E (APOE) ε4 carriers and Aβ deposition on PET were higher in the ADD group than in the OND and CU group. There was a significant difference in the Clinical Dementia Rating (CDR) scale among the groups. The Mini-Mental State Examination (MMSE)^24^ and CDR-Sum of Boxes (SB) scores were significantly different among the groups after adjusted for age. The MMSE (*p* < 0.001), and CDR-SB (*p* < 0.001) scores were significantly lower in the ADD group than in the CU group. The MMSE (*p* = 0.003) and CDR-SB (*p* < 0.001) scores were also significantly lower in the ADD group than in the OND group. Finally, the MMSE (*p* < 0.001) and CDR-SB (*p* < 0.001) scores were significantly lower in the OND group than in the CU group.

**Table 1:** Demographic and clinical characteristics of the participants. Notes: ADD = Alzheimer’s disease dementia; OND = other neurological disorders; CU = cognitively unimpaired; MMSE = Mini-Mental State Examination; CDR = Clinical Dementia Rating scale; CDR-SB = CDR-Sum of Boxes; APOE = apolipoprotein E; Aβ, amyloid beta. Values are mean (standard deviation) or numbers (%).

Variables	ADD (n = 35)	OND (n = 18)	CU (n = 26)	*P* value	*P* < 0.05^§^
Age, years	75.8 (9.9)	76.9 (7.5)	68.9 (5.7)	0.002^*^	b, c
Female	26 (74.3%)	12 (66.7%)	22 (84.6%)	0.373^†^	
Education, years	7.4 (5.0)	7.0 (4.6)	6.7 (4.8)	0.861^*^	
MMSE	15.2 (3.7)	18.9 (5.5)	26.1 (2.8)	<0.001^‡^	a, b, c
CDR 0: 0.5: 1: 2: 3	0: 12: 15: 7: 1	0: 12: 4: 2: 0	26: 0: 0: 0: 0	<0.001^†^	
CDR-SB	6.34 (4.05)	3.94 (3.67)	0.19 (0.29)	<0.001^‡^	a, b, c
APOE ε4 carrier	12/33 (36.4%)	1/13 (7.7%)	3/22 (13.6%)	0.041^†^	
Aβ deposition on PET	28/28	0/15	0/20	<0.001^†^	

*P value from analysis of variance; ^†^Results from chi-square tests; ^‡^*P* value from analysis of covariance with age as a covariate; ^§^Pairwise comparisons on age between diagnosis groups were conducted with Tukey method, and pairwise comparisons on MMSE and CDR-SB between diagnosis groups were conducted with Bonferroni post hoc analysis; a, ADD vs. OND; b, ADD vs. CU; c, OND vs. CU.

**Figure 1:**
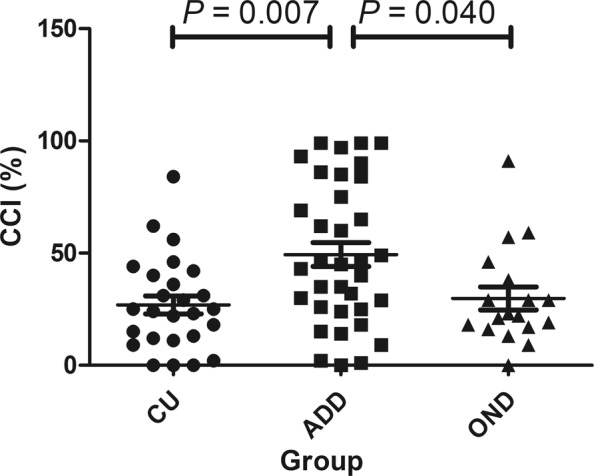
The distribution pattern of the capacitance change index (CCI) in nasal secretions in patients with Alzheimer’s disease dementia (ADD), those with cognitive decline related to other neurological diseases (OND), and cognitively unimpaired (CU) individuals. The CCI was significantly higher in the ADD group than in the OND (*p* = 0.040) and CU groups (*p* = 0.007) after application of Bonferroni post hoc analysis.

The capacitance change index (CCI) in nasal secretions was significantly different among the groups after adjusted for age (Table 2). Bonferroni post hoc analysis showed that the CCI in nasal secretions was significantly higher in the ADD group than in the OND (*p* = 0.040) and CU groups (*p* = 0.007) (Fig. 1). The CCI in nasal secretions showed a negative correlation with the MMSE score and a positive correlation with CDR or CDR-SB scores (Table 3).

**Table 2:** Capacitance change index in nasal secretions according to each group.

Variables	ADD (n = 35)	OND (n = 18)	CU (n = 26)	*P* value*	Pairwise comparisons^†^
CCI	49.4 (31.7)	29.8 (21.8)	26.9 (20.6)	0.003	P_a_ = 0.040 P_b_ = 0.007 P_c_ = 1.000

Notes: ADD = Alzheimer’s disease dementia; OND = other neurological disorders; CU = cognitively unimpaired; CCI = capacitance change index. Values are mean (standard deviation). *P value from analysis of covariance with age as a covariate; ^†^Results from Bonferroni post hoc analysis; a, ADD vs. OND; b, ADD vs. CU; c, OND vs. CU.

**Table 3:** Correlations between the capacitance change index and global cognitive function. Notes: MMSE = Mini-Mental State Examination; CDR = Clinical Dementia Rating scale; CDR-SB = CDR-Sum of Boxes.

	Capacitance change index	
	Spearman’s rho	*P* value
MMSE (range, 0–30)*	−0.252	0.027
CDR (range, 0–3)^†^	0.271	0.016
CDR-SB (range, 0–18)^†^	0.294	0.009

*Decreases in scores represent worsening. ^†^Increases in scores represent worsening.

**Figure 2:**
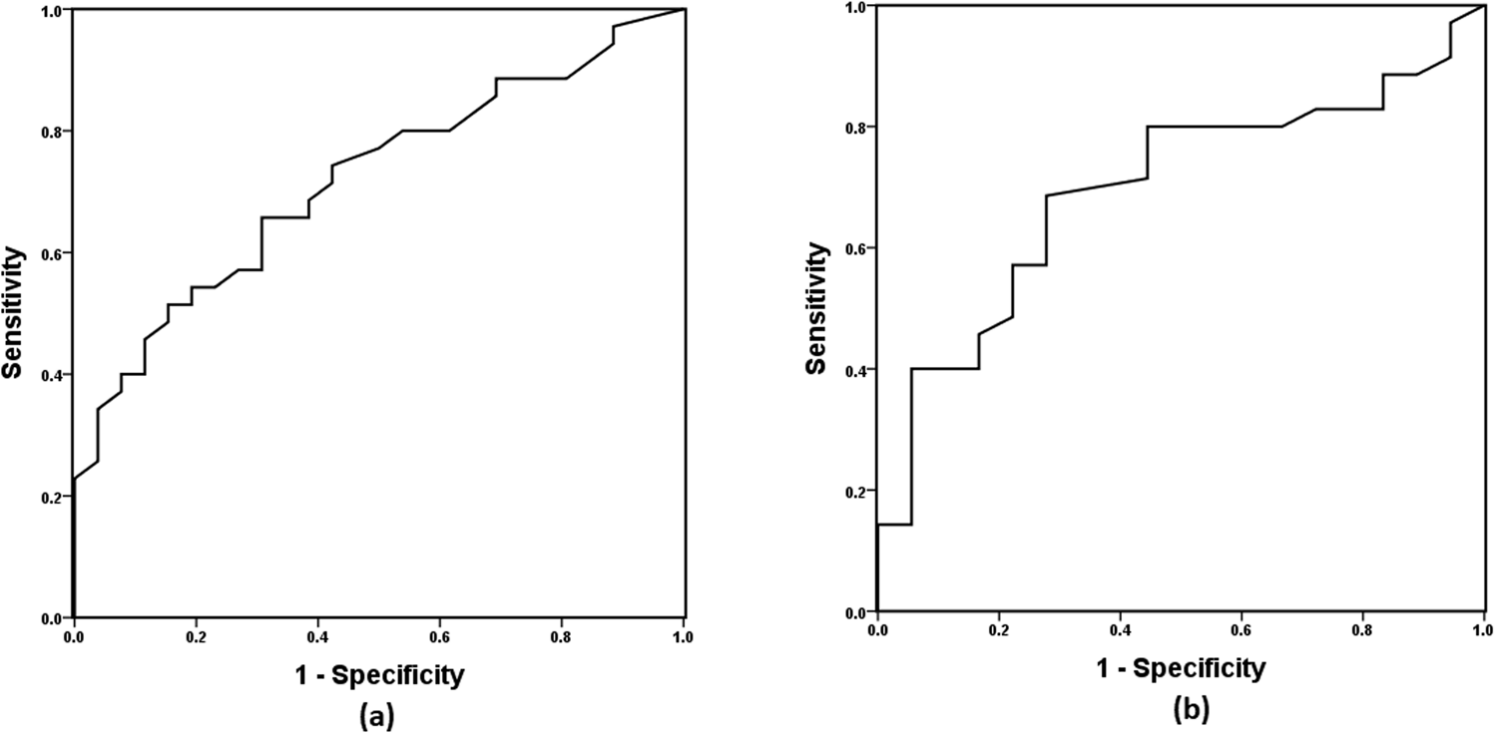
The receiver operating characteristic (ROC) curve of the capacitance change index (CCI) used for the diagnosis of Alzheimer’s disease dementia (ADD). (**a**) The sensitivity and specificity of the CCI for the diagnosis of ADD among patients with ADD and cognitively unimpaired individuals was 65.7% and 69.2%, respectively, at the cutoff point of 31.5. The area under the ROC curve (AUC) was 0.718 (95% confidence interval [CI], 0.591–0.845, standard error [SE] = 0.065, *p* = 0.004). (**b**) The sensitivity and specificity of the CCI in nasal secretions for the diagnosis of ADD among participants with ADD or other neurological disorders was 68.6% and 72.2%, respectively, at the cutoff point of 29.5. The AUC was 0.696 (95% CI, 0.551–0.841, SE = 0.074, *p* = 0.020).

The receiver operating characteristic (ROC) curve was used to examine the predictive value of the CCI in nasal secretions for a diagnosis of ADD. The area under the ROC curve (AUC) was 0.718 (95% confidence interval [CI], 0.591–0.845, standard error [SE] = 0.065, *p* = 0.004) for the diagnosis of ADD among the participants of the ADD and CU groups (Fig. 2a). The sensitivity and specificity values of the CCI in nasal secretions in the diagnosis of ADD were 65.7% and 69.2%, respectively, at the cutoff point of 31.5. The ability of the CCI in nasal secretions to differentiate between ADD and OND patients was newly validated in a separate ROC analysis. The AUC was 0.696 (95% CI, 0.551–0.841, SE = 0.074, *p* = 0.020) for the diagnosis of ADD among the participants of the ADD and OND groups (Fig. 2b). The sensitivity and specificity values of the CCI in nasal secretions for a diagnosis of ADD among the participants of the ADD and OND groups were 68.6% and 72.2%, respectively, at the cutoff point of 29.5.

**Figure 3:**
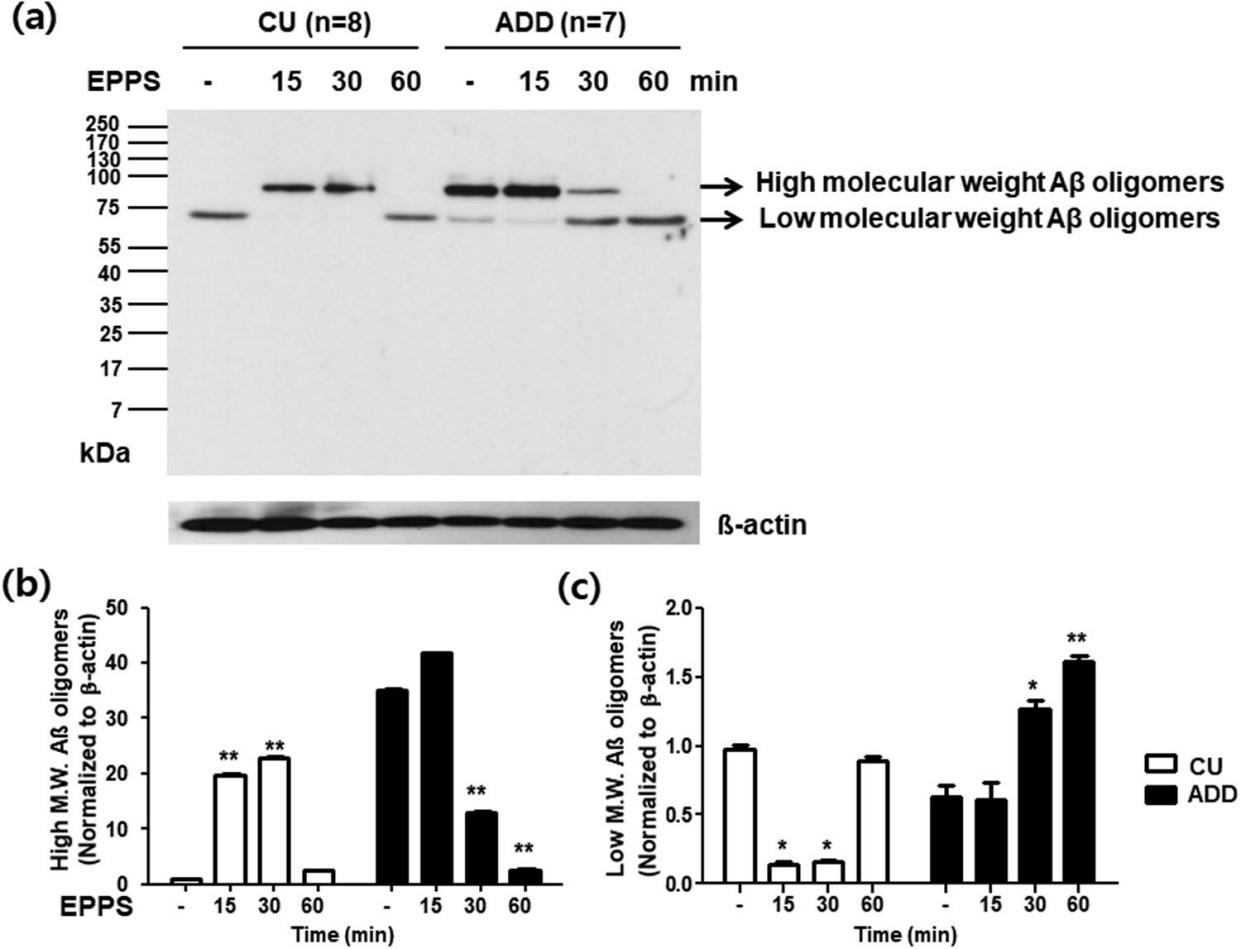
Western blot analysis of nasal secretions in 7 patients with Alzheimer’s disease dementia (ADD) and 8 cognitively unimpaired (CU) individuals. Western blots using 6E10 antibody were performed for nasal secretions of 7 ADD patients with amyloid deposition on PET and 8 CU individuals without amyloid deposition on PET. (**a**) Western blotting of nasal secretions of the ADD patients and CU individuals at baseline, and 15, 30 and 60 min after 4-(2-hydroxyethyl)-1-piperazinepropanesulfonic acid (EPPS) treatment. It presents that the baseline nasal secretion of ADD patients contains larger amount of high molecular weight amyloid β (Aβ) oligomers of ~90 kDa compared to the CU individuals and less other molecular weight Aβ oligomers (lane 1, 5), and by treating EPPS for 60 min, high molecular weight Aβ oligomers were gradually disassembled to lower molecular weight Aβ oligomers under 70 kDa. After 60 min, a band at ~90 kDa completely disappeared and a band at ~70 kDa definitely emerged (lane 6–8). (**b**) The amount of high molecular weight Aβ oligomers gradually decreases until 60 min in the nasal sample of ADD patients. The amount of high molecular weight Aβ oligomers in the ADD group was significantly lower at 30 min and 60 min than at baseline. (**c**) The amount of disassembled lower molecular weight Aβ oligomers gradually increases until 60 min in the nasal sample of ADD patients. The amount of disassembled lower molecular weight Aβ oligomers in the ADD group was significantly higher at 30 min and 60 min than at baseline. Data are mean (SEM). *p < 0.005 vs. baseline and **p < 0.001 vs. baseline by Bonferroni post hoc analysis.

To confirm the disassembly of Aβ oligomers related to the principle of capacitance change of IME sensor, the nasal secretion samples of 7 ADD patients and 8 CU individuals at baseline and their 4-(2-hydroxyethyl)-1-piperazinepropanesulfonic acid (EPPS)-treated samples according to time (15, 30, and 60 min) were analyzed by western blots. Figure 3a presents that the baseline nasal secretions of ADD patients contain larger amounts of high molecular weight Aβ oligomers of ~90 kDa compared to the CU individuals and less other molecular weight Aβ oligomers (lane 5), and by treating EPPS for 60 min, high molecular weight Aβ oligomers were gradually disassembled to lower molecular weight Aβ oligomers with a distinct band at ~70 kDa and various sizes of Aβ oligomers under 70 kDa. After 60 min, a band at ~90 kDa completely disappeared and a band at ~70 kDa definitely emerged (lane 6–8). The amount of high molecular weight Aβ oligomers in the ADD group was significantly lower at 30 min and 60 min than at baseline (Fig. 3b). The amount of disassembled lower molecular weight Aβ oligomers gradually increased until 60 min in the nasal sample of ADD patiens, and the amount of disassembled lower molecular weight Aβ oligomers in the ADD group was significantly higher at 30 min and 60 min than at baseline (Fig. 3c).

## Discussion

In this study, the CCI in nasal secretions was elevated in the patients with ADD compared to the CU individuals and those with OND. The CCI in nasal secretions is greater when the level of soluble Aβ in nasal secretions is higher. Therefore, the present results demonstrate that the soluble Aβ such as oligomeric Aβ in nasal secretions is elevated in ADD patients compared to CU individuals and those with OND. The present results demonstrate the possibility that the ultra-sensitive detection of soluble Aβ in nasal secretions using IME biosensors may be effective in predicting ADD. However, the accuracy of the CCI as measured by the AUC was fair in separating into the ADD and CU groups. The accuracy of CCI in nasal secretions for the prediction of ADD was lower than that of amyloid PET ligands and CSF AD biomarkers found in previous studies^11,25^. More studies are needed before Aβ in nasal section can be used as a biomarker for AD. The following studies may be needed to improve accuracy. First, the amount of nasal secretions collected varied among the participants, so the concentration of Aβ needs to be considered. Second, if there is a diurnal variation in the amount of Aβ in nasal secretions, it is necessary to collect them from participants at similar times. Third, we need to consider using a more powerful substance than EPPS to disaggregate high molecular weight Aβ. Fourth, more advanced measurement system may be needed.

The accuracy of the CCI in nasal secretions was also nearly fair to distinguish between ADD and OND patients. To be a diagnostic marker for ADD, it is important that a biomarker is able to distinguish ADD from patients with other diseases as well as from normal individuals. Although the results of this study are still lacking, the present results suggest that if accuracy improves, the CCI reflecting the amount of soluble Aβ in nasal secretions may be useful in distinguishing ADD from other causes of dementia. Although the presence or absence of amyloid deposition using PET or CSF studies was found in about 82% of the participants in this study, some participants of the OND group may also have had multiple brain pathologies including AD^26^. In the future, the CCI should be evaluated only in OND patients with amyloid-negative results on PET or CSF studies.

The CCI was significantly correlated with global cognitive function scales. Higher CCI was related to lower global cognitive function, and thus the CCI may reflect the natural course of AD. However, whether a change in the CCI is associated with cognitive decline should be evaluated in a longitudinal observational study. No correlation was observed between CSF and PET amyloid value changes in a longitudinal study^27^. This result suggests that Aβ in body fluids may measure different aspects of AD Aβ pathology from those of amyloid PET.

The ultra-sensitive detection of small amounts of Aβ in nasal secretions could be performed using IME methods^28^. Previously, we had failed to measure Aβ in nasal secretions by Luminex multiplex assay. Electrical impedimetric biosensors have attracted attention due to their label-free and real-time monitoring of the morphological and physiological changes in DNA, protein, and cells^29^. For the electrical impedance measurement of the specimens, micro- or nano-sized electrode-based sensors are used. The IME biosensor could therefore be a method by which ultra-low levels of proteins are measured in body fluids.

The previous clinical studies and a meta-analysis showed that patients with ADD had the evidence for olfactory deficits^30,31^. In a previous report, olfactory impairment in cognitively normal elderly was associated with incident amnestic mild cognitive impairment (aMCI) and progression from aMCI to ADD over a mean 3.5 years of follow-up^32^. This study suggests that olfactory deterioration may precede cognitive decline in AD. More research is needed on the olfactory function and measurement of Aβ in nasal secretions in AD.

The study of EPPS-derived disassembly of soluble Aβ by the western blots demonstrated that soluble Aβ proteins such as high molecular weight Aβ oligomer^33^ were disassembled to several numbers of lower molecular weight Aβ oligomers (~70 kDa and much smaller oligomers). Thus, it looks clear that the increase of target molecules is closely related to the increase of capacitance change (ΔC_oAβ_) and furthermore, clinical effectiveness of CCI for ADD diagnosis based on the nasal secretions. The reason why other bands of disassembled Aβ oligomers except a band at ~70 kDa were not clearly shown in the blot image might have been that their concentrations were too low to be detectable by western blots. The western blot analysis did not provide any information on the isoforms of Aβ oligomers (40 and 42 or their mixture) captured by 6E10 anti-Aβ antibody as well. Nevertheless, Aβ_42_ can be the most reasonable candidate among the amyloid proteins which are contained in discrete bands. In a previous study, authors have used a western blot analysis, to which 6E10 antibody applied, for the characterization of high molecular weight Aβ_42_ oligomers as well as its monomer isolated from human hippocampal samples^34^.

Our study had several limitations. First, the results should be interpreted with caution due to the small sample size. The results of this study should be reproduced in other studies with large sample sizes. Second, amyloid biomarkers such as amyloid PET ligands and CSF biomarkers were not studied for all participants, although approximately 82% of participants had historical results available for amyloid PET or CSF Aβ_42_ levels. Some patients without brain amyloid pathology may have been included in the ADD group, while other individuals with brain amyloid pathology may have been included in the CU and OND groups. Third, we did not examine the associations of the CCI in nasal secretions with other AD biomarkers such as amyloid PET, CSF Aβ_42_, t-tau and p-tau levels, and cortical thickness. Fourth, we did not directly measure the levels of Aβ in nasal secretions, but rather, we measured the Aβ levels in nasal secretions indirectly by assessing changes in capacitance. Fifth, we did not pinpoint the species of Aβ detected by 6E10 anti-Aβ antibody in the baseline nasal secretions as well as in the nasal secretions after disaggregation by EPPS. In the future, it is necessary to define the size and isoforms of Aβ using mass spectrometry. It is also necessary to analyze nasal secretion with other anti-Aβ antibodies, such as antibodies targeting oligomeric Aβ_42_. Sixth, we did not investigate the efficiency of examining the amount of EPPS that allows the CCI to reach its maximum value^35^.

To our knowledge, this is the first study to show that Aβ levels in nasal secretions are elevated in ADD patients compared to CU individuals and those with OND. The results of this study are still preliminary but suggest that CCI reflecting the amount of soluble Aβ in nasal secretions, such as oligomeric Aβ, may be useful in predicting ADD if accuracy improves. More studies are needed before Aβ in nasal secretions can be used as a biomarker for AD.

## Methods

### Participants

From October 2015 to August 2018, 38 patients with ADD, 18 patients with cognitive decline associated with OND, and 27 CU elderly controls participated in this study. Three patients with ADD and one CU individual withdrew their consent. Finally, nasal secretions were obtained from 79 participants, who ranged in age from 50 to 90 years. We recruited patients with ADD or OND from the memory clinic at Inha University Hospital, Incheon, South Korea. The participants with ADD met the criteria for probable ADD according to the National Institute on Aging-Alzheimer’s Association core clinical criteria^36^. The OND group consisted of nine patients with vascular cognitive impairment^37^, three patients with frontotemporal lobar degeneration^38^, one patient with Parkinson’s disease^39^, one patient with epilepsy, and four patients with other dementia.

The CU individuals were recruited from patients who visited the neurology clinic at the same hospital for other reasons (e.g., dizziness, hypertension, Bell’s palsy). They did not have any of the 28 diseases associated with decreases in cognitive function or a history suggestive of a decrease in cognitive function^40^: stroke or transient ischaemic attack, seizures, Parkinson’s disease, multiple sclerosis, cerebral palsy, Huntington’s disease, encephalitis, meningitis, brain surgery, surgery to clear arteries to the brain, diabetes that requires insulin to control, hypertension that is not well controlled, cancer other than skin cancer diagnosed within the past three years, shortness of breath while sitting still, use of oxygen at home, heart attack that resulted in changes in memory, walking or problem solving lasting at least 24 hours, kidney dialysis, liver disease, hospitalization for mental or emotional problems within the past five years, current use of medications for mental or emotional problems, alcohol consumption greater than three drinks per day, drug abuse in the past five years, treatment for alcohol abuse in the past five years, unconsciousness for more than one hour other than during surgery, overnight hospitalization because of a head injury, illness causing a permanent decrease in memory or other mental functions, trouble with vision that prevents reading ordinary print even with glasses, and difficulty understanding conversations because of hearing even if wearing a hearing aid. They also had MMSE scores more than 1.5 standard deviation below the age- and education adjusted normative means^24^.

We excluded participants who took medications that affect nasal function within 1 week of the study entry, those who underwent nasal surgery within 3 months, those who were diagnosed with rhinitis or sinusitis by simple X-ray or computed tomography scan, those with a history of exposure to chemical stimulants, current smokers, those with severe or uncontrolled medical disease, and those who were pregnant or lactating.

All participants underwent comprehensive physical and neurological examinations, including an extensive standardized neuropsychological battery, the Seoul Neuropsychological Screening Battery (SNSB)^41^. They were also administered a brain MRI and routine biochemical and serological tests that included a thyroid function test, tests for serum vitamin B12 and folate levels, and a Venereal Disease Research Laboratory test.

The presence or absence of cortical amyloid deposition was confirmed by amyloid PET imaging in 63 (79.7%) participants and by CSF Aβ measurements in two participants. Evidence of brain amyloid pathology in the ADD group was demonstrated in fifteen patients with positive ^18^F-flutemetamol PET imaging, nine with positive ^18^F-florbetaben PET imaging, three with positive ¹¹C-Pittsburgh compound-B (^11^C-PIB) PET imaging, and one with positive ^18^F-florbetapir PET imaging (Table 1). Amyloid pathology in the brain was demonstrated by low Aß_42_ levels in the CSF in two other patients in the ADD group. There were eight participants with amyloid-negative results on ^18^F-flutemetamol PET imaging and seven with amyloid-negative results on ^18^F-florbetaben PET imaging in the OND group. There were ten participants with amyloid-negative results on ^18^F-flutemetamol PET imaging, nine with amyloid-negative results on ^18^F-florbetaben PET imaging, and one with amyloid-negative results on ^11^C-PIB PET imaging in the CU group. The amyloid PET data were visually assessed by two doctors of nuclear medicine, who were masked to the clinical diagnosis and all other clinical findings.

The study was performed in accordance with the International Harmonization Conference guidelines on Good Clinical Practice and was approved by the institutional review board of Inha University Hospital prior to study initiation. Prior to participation in the study, all participants provided written informed consent for their participation in the study. The legal representatives of the participants with dementia also provided written informed consent.

### Collection of nasal secretions

The collection of nasal secretions was performed according to the method previously reported by Cho *et al*.^42^ A Merocel^®^ sponge was placed into both nasal cavities of the patient for 1 minute. At this stage, the sponge was placed towards the nasal roof so that it was as close to the olfactory bulb as possible. After the sponge was removed from the participant’s nose, we inserted it in a specially shaped tube with a funnel. When the sponge is placed on the funnel in this tube, the nasal secretion absorbed by the sponge flows down through the funnel which is designed to be tucked down. We performed centrifugation at 4 °C and 5000 rpm for 10 minutes to isolate all nasal secretions from the sponge. The nasal secretions were then stored at a temperature of −80 °C until further analysis.

### System Configuration of AD Diagnostic System

**Figure 4:**
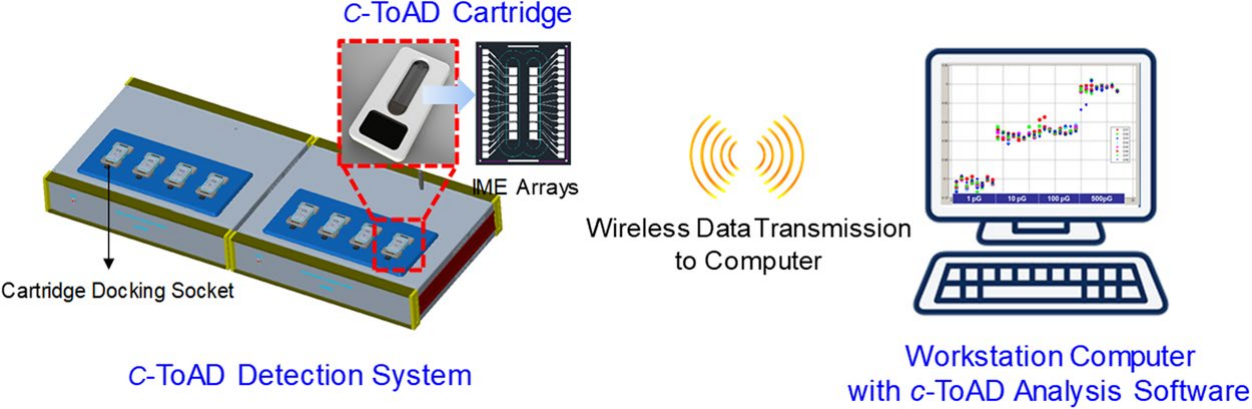
Overview of the *c*-ToAD diagnostic system developed by Cantis Inc. IME = interdigitated microelectrode.

The electrical capacitance measurement-based AD diagnostic system (*c-*ToAD, Cantis Inc., South Korea) used to analyse human nasal secretions is briefly composed of (i) *c*-ToAD detection system, which is a customized impedance analyser with 8 device docking sockets and a syringe pump, (ii) the workstation computer for data acquisition from the *c*-ToAD detection system, the process of which is related to visualization and motion control of the syringe pump connected to the *c*-ToAD cartridge, and (iii) the disposable *c*-ToAD cartridge. All communications between the *c*-ToAD detection system including the micro-pump and the computer were achieved via Bluetooth wireless technology (Fig. 4). In detail, the cartridge is composed of a glass chip on which 16 interdigitated platinum microelectrode (Pt-IME) arrays were fabricated, polycarbonate (PC) packaging with 2 microfluidic channels containing 8 electrodes per channel and the electric interface that connects Pt-IMEs to the device docking sockets. To analyse the human samples, IgG monoclonal antibody 6E10 (BioLegend, USA) used to capture Aβ was immobilized between Pt-IMEs in the ToAD cartridges, which were prepared immediately before use.

### Sample Analysis Protocol

**Figure 5:**
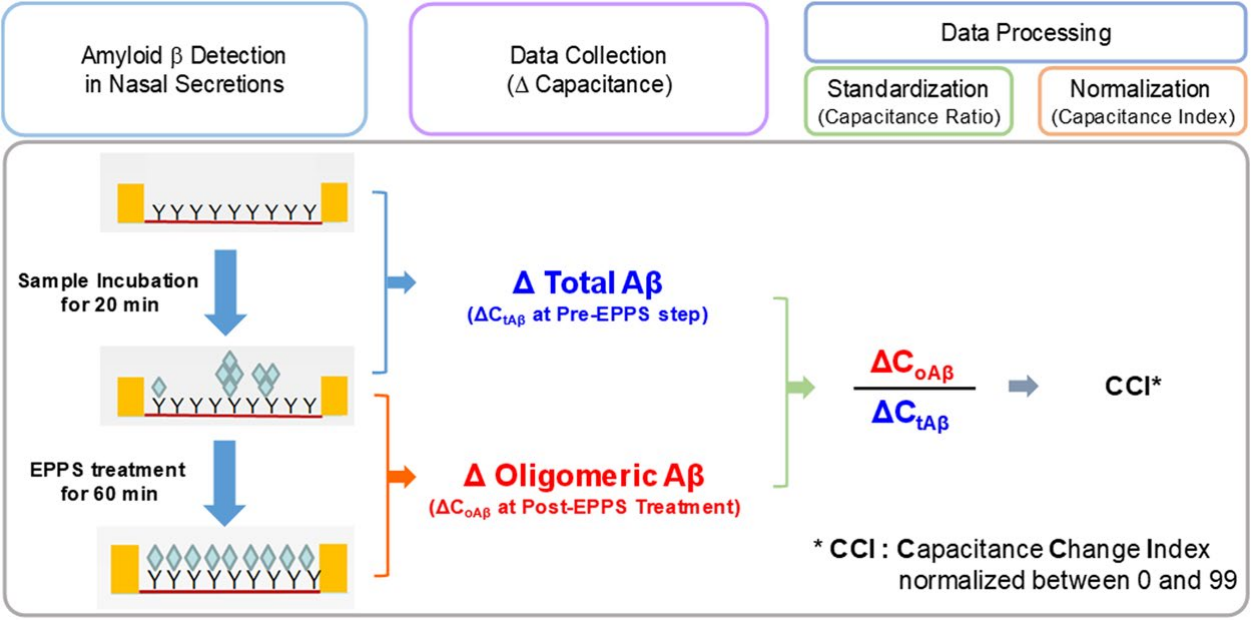
Assay protocol for the measurement of oligomeric amyloid β in diluted nasal secretions. It shows the process by which electronic data are collected from the ToAD diagnostic system and data standardization/normalization through which the capacitance change index is calculated. EPPS = 4-(2-hydroxyethyl)-1-piperazinepropanesulfonic acid.

Before the analyse, the nasal secretions were collectively diluted to 25% of their original concentration in phosphate-buffered saline (PBS) (1×, pH 7.4) to adjust their viscosity, after which they were contained in a 200 μL syringe. By driving the microsyringe pump, a maximum of 16 samples was carefully delivered to the microfluidic channels of the *c*-ToAD cartridges. The samples were then incubated for 20 min at room temperature. To clean the IMEs and channels, the sample solutions were pumped out, and the microfluidic channels were washed with PBS with Tween 20 (×1) and PBS (×3) sequentially by repeating the flow of the wash buffers. Capacitance changes in the IMEs were measured by capturing the molecules of total Aβ at this step (pre-EPPS step, 1000 Hz, 20 min). After the measurement, EPPS was carefully injected into the microfluidic channels, and simultaneously, the second capacitance measurement was performed (1000 Hz, 1 h). The capacitance change over time at the post-EPPS step indicates that monomeric or lower molecular weight Aβ molecules that disassembled from oligomeric Aβ by EPPS were gradually associated with the unoccupied antigen binding fragment (Fab) binding sites of the Aβ antibody (Fig. 5)^33^. Using the capacitance change, the system was able to avoid the deviation of the capacitance values of the IME array that were determined by the different lengths and locations of the transmission lines between the sensing area and the terminal pads.

### Data analysis

As mentioned above, all data measured by the *c*-ToAD detection system were spontaneously transmitted to the *c*-ToAD workstation via wireless transmission during measurement. In principle, the *c*-ToAD software averaged all of capacitances obtained from the 8-IME array in a single microfluidic channel according to an electric parameter setting and algorithm and calculated capacitance changes of the total Aβ (ΔC_tAβ_) at the pre-EPPS step and those of the oligomeric Aβ (ΔC_oAβ_) at the post-EPPS step to yield the capacitance ratio (ΔC_oAβ_/ΔC_tAβ_) of each sample. Finally, all of ΔC_oAβ_/ΔC_tAβ_ values were normalized to specific indices between 0 and 99 according to a measurement standard (Capacitance Change Index, CCI (a.u.)).

### Western blot analysis of nasal secretions

Nasal secretions collected from 7 ADD patients with amyloid deposition on PET and 8 CU individuals without amyloid deposition on PET were used in the western blot analysis. Nasal secretions were lysed in ice-cold RIPA buffer containing 1× proteinase inhibitor cocktail. Lysate was incubated in ice for 20 minutes and centrifuged at 14,000 r.p.m., 4 °C for 30 min. The supernatant was used in Aβ western blots for biochemical changes. Protein samples (40 μg) were loaded on each lane of SDS–PAGE gels to detect Aβ oligomer, disassembled lower molecular Aβ oligomer and β-actin (a loading control). Afterward, the blots were probed with anti-Aβ (6E10) or anti-β-actin antibodies (1:1000) at 4 °C overnight, followed by incubation with secondary goat anti-mouse horse radish peroxidase-conjugated immunoglobulin G (1:20,000) at room temperature for 1 h. The signals were visualized using enhanced chemiluminescence. The band intensity was quantified on ImageJ software (http://rsbweb.nih.gov/ij/index.html).

### Statistical analyses

Characteristic data were presented as means and standard deviations (SD) for continuous variables and frequency and percentages for categorical variables. The analysis of variance (ANOVA) was used to compare age and education among the groups. When a statistical significant overall difference was detected in the ANOVA test, pairwise comparisons on means between diagnosis groups were conducted with Tukey method. For the categorical variables, namely, gender, CDR, APOE ε4 carrier status and Aβ deposition on PET, we calculated the frequencies and compared their differences across the groups using the χ² test. The analysis of covariance (ANCOVA) with age as a covariate was used to compare MMSE, CDR-SB, and CCI among the groups. When a statistical significant overall difference was detected in the ANCOVA test, pairwise comparisons on means between diagnosis groups were conducted with Bonferroni post hoc analysis. The ANOVA and Bonferroni post hoc analysis were used to compare the amounts of proteins according to the time in the western blot. Spearman’s rho was used to measure the correlations between CCI and global cognitive function. The sensitivity and specificity of the CCI for the diagnosis of ADD were evaluated by ROC curve analysis. The cutoff values were determined to yield the best Youden index (sensitivity + specificity − 1) for ADD diagnosis. Significance for all tests was set at α = 0.05, two-tailed. All statistical analyses were performed using SPSS 19.0 (SPSS, Chicago, IL, USA).

## Supplementary information

Supplementary Information

## Data Availability

Data is available.
