# Supplementary material for: Amyloid beta in nasal secretions may be a potential biomarker of Alzheimer’s disease
Source: Sci Rep. 2019 Mar 21;9:4966. doi: 10.1038/s41598-019-41429-1 (PMC6428828; doi:10.1038/s41598-019-41429-1)
Supplement: Supplementary file 1 — Supplementary Information [file 41598_2019_41429_MOESM1_ESM.pdf]

Amyloid beta in nasal secretions may be a potential biomarker of Alzheimer's disease

Young Hyo Kim,[1*] Sang-Myung Lee,[2*] Sungbo Cho,[3] Ju-Hee Kang,[4] Yang-Ki Minn,[5] Hyelim Park,[1] Seong Hye Choi[6]

(*These authors contributed equally to this work.)

[1]Departments of Otorhinolaryngology-Head and Neck Surgery, Inha University School of Medicine, Incheon22332, South Korea; [2]Department of Chemical Engineering, Kangwon National University, Chuncheon24341, South Korea; [3]Department of Electronic Engineering, Gachon University, Seongnam13120, South Korea; [4]Department of Pharmacology, Inha University School of Medicine, Incheon22212, South Korea; [5]Department of Neurology, Hallym University Kangnam Sacred Heart Hospital, Hallym University College of Medicine, Seoul07441, South Korea; [6]Department of Neurology, Inha University School of Medicine, Incheon22332, South Korea

Corresponding author: Seong Hye Choi MD, PhD

Department of Neurology, Inha University Hospital

27 Inhang-ro, Jung-gu, Incheon 22332, South Korea

Tel: 82 32 890 3860

FAX: 82 32 890 1140

E-mail: seonghye@inha.ac.kr

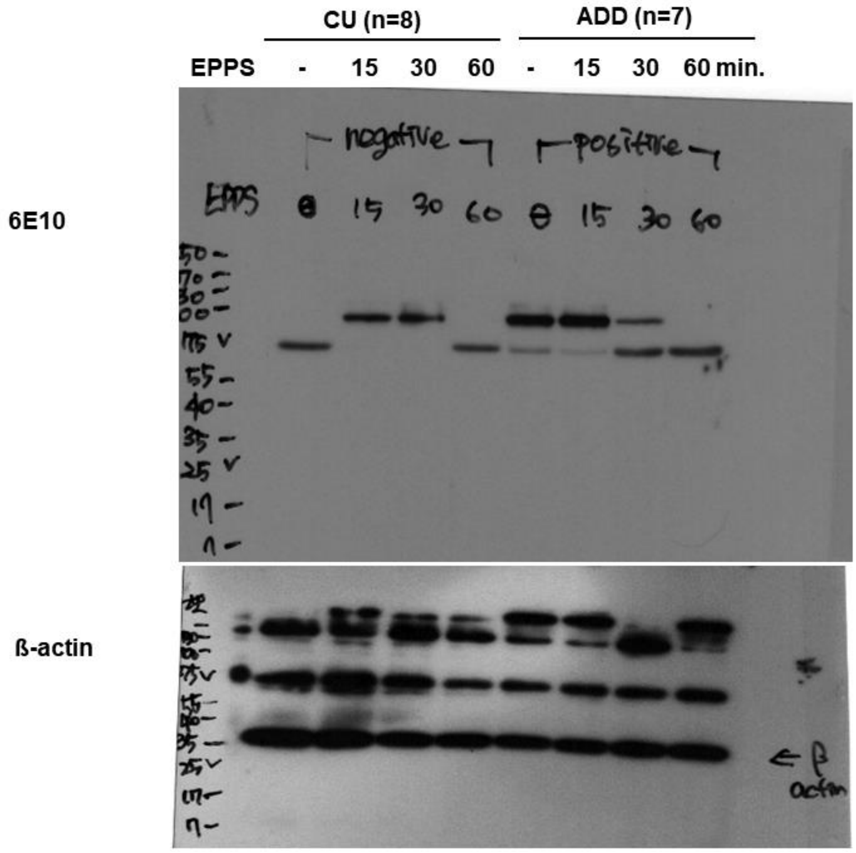
CU (n=8)
ADD (n=7)
EPPS - 15 30 60 - 15 30 60 min.
6E10
ß-actin
